# "Othering" the health worker: self-stigmatization of HIV/AIDS care among health workers in Swaziland

**DOI:** 10.1186/1758-2652-14-60

**Published:** 2011-12-22

**Authors:** Daniel H de Vries, Shannon Galvin, Masitsela Mhlanga, Brian Cindzi, Thabsile Dlamini

**Affiliations:** 1 Department of Sociology and Anthropology, Amsterdam Institute for Social Science Research, University of Amsterdam, Amsterdam, the Netherlands; 2 Northwestern University, Division of Infectious Disease, Michigan Avenue, Chicago, USA; 3 Swaziland Nurses Association, PO Box 2031, Manzini, Swaziland

## Abstract

**Background:**

HIV is an important factor affecting healthcare workforce capacity in high-prevalence countries, such as Swaziland. It contributes to loss of valuable healthcare providers directly through death and absenteeism and indirectly by affecting family members, increasing work volume and decreasing performance. This study explored perceived barriers to accessing HIV/AIDS care and prevention services among health workers in Swaziland. We asked health workers about their views on how HIV affects Swaziland's health workforce and what barriers and strategies health workers have for addressing HIV and using healthcare treatment facilities.

**Methods:**

Thirty-four semi-structured, in-depth interviews, including a limited set of quantitative questions, were conducted among health workers at health facilities representing the mixture of facility type, level and location found in the Swaziland health system. Data were collected by a team of Swazi nurses who had received training in research methods. Study sites were selected using a purposive sampling method while health workers were sampled conveniently with attention to representing a mixture of different cadres. Data were analyzed using Nvivo qualitative analysis software and Excel.

**Results:**

Health workers reported that HIV had a range of negative impacts on their colleagues and identified HIV testing and care as one of the most important services to offer health workers. They overwhelmingly wanted to know their own HIV status. However, they also indicated that in general, health workers were reluctant to access testing or care as they feared stigmatization by patients *and *colleagues and breaches of confidentiality. They described a self-stigmatization related to a professional need to maintain a HIV-free status, contrasting with the HIV-vulnerable general population. Breaching of this boundary included feelings of professional embarrassment and fear of colleagues' and patients' judgements.

**Conclusions:**

While care is available and relatively accessible, Swaziland health workers still face unique usage barriers that relate to a self-stigmatizing process of boundary maintenance - described here as a form of "othering" from the HIV-vulnerable general population - and a lack of trust in privacy and confidentiality. Interventions that target health workers should address these issues.

## Background

The HIV pandemic does not spare health workers. In some areas particularly hit hard by AIDS, such as Zambia's Lusaka and Kasama districts, annual death rates of 3.5% for nurses and 2.8% for clinical officers have been claimed [1]. For Swaziland, the annual mortality among health workers due to HIV/AIDS was 5% in 2004 [2]. HIV prevalence among health staff has been reported to equal the general population - 26% among adults aged 15-49 years [3,4] - and HIV-related death rates have risen to 4% annually [5].

In neighbouring South Africa, the HIV prevalence among professional healthcare workers was found to range from 12.2% to 19.9% [6]. Over the coming years, sub-Saharan African health systems may lose up to one-fifth of their employees to HIV/AIDS [3]. This attrition may have a severe impact on regional human resources for health capacity, leaving critical efforts, such as the general roll out of antiretroviral therapy (ART), barely feasible [7,8]. However, while health workers tend to know where to go to obtain an HIV test, reluctance to test and low access to post-exposure prophylaxis (PEP) has been found in the literature [9-11].

What barriers may exist that prevent caregivers from accessing needed care? One overall finding has been an emphasis on the role of stigma on usage of special HIV/AIDS services by health workers [12-14]. Health workers fear that if they disclose their HIV-positive status or if they have to queue alongside their patients for treatment, patients will lose confidence in them as they will be perceived as sinful and unable to follow their own prevention messages [3,15]. Some health workers fear that this loss of authority could lead to loss of patients, impact their social status [15], and affect their employment security [16]. This negative attitude toward people living with HIV (PLHIV) appears to be not restricted to patients and the larger community, but is also prevalent among professional health workers through charting, labelling, gossip, verbal harassment, avoidance, isolation and referrals for testing [9,17,18]. An issue complicating stigma toward PLHIV is a lack of knowledge among both patients and providers about modes of HIV transmission [17].

Analyses focusing on health worker access to care are only recently emerging. A lack of well-established HIV infection treatment programmes targeting health workers makes comparative knowledge about the optimal methods hard to find. HIV care, integrated with other comprehensive services in staff clinics located in house or in stand-alone services close to the hospital, has shown positive utilization results [12]. However, it also has been observed that many health workers prefer to seek care far away from where they live or work, which means incurring considerable extra financial costs [14]. Outside the health worker context, in-house (employer) and independent disease management have achieved higher uptake of services than medical aid schemes, but overall usage has remained low [19].

To better understand low utilization of HIV/AIDS services for health workers and develop recommendations, a participatory study was designed to document perceived barriers to accessing HIV/AIDS care and prevention services among health workers in Swaziland. The study was implemented by a core group of nurses from the Swaziland Nursing Association (SNA) in collaboration with the Southern Africa Human Capacity Development Coalition, the Swaziland Ministry of Health and Social Welfare (MOHSW) and USAID's Capacity Project, led by IntraHealth International.

## Methods

### Study design

The study used a participatory, qualitative research method with a small survey component. Nurses working in Swaziland were included as interviewers and analysts.

A semi-structured, primarily open-ended interview questionnaire was used. It included a limited set of quantitative question using binary answers, never/sometimes/always scales, and forced-choice likert scales. Barriers to HIV treatment were depicted via the use of two vignettes (scenarios) based on actual situations reported by SNA nurses [20]. The vignettes were used to circumvent direct questions about a respondent's HIV/AIDS status - to protect privacy, we did not want to directly ask if respondents had been tested and were HIV positive - and ensure acceptability of the study [21].

### Sampling and data collection

Relatively senior nurses of the SNA conducted interviews after receiving professional training on qualitative interviewing methods. Questionnaires were translated into both siSwati and English and administered in the language preferred by the respondent. Data were collected over a seven-week period in October and November 2007 in nine health facilities located in all four Swaziland regions. Purposive sampling was used in the identification of the first eight health facilities choosing maximum variation across area type (urban, rural, company), ownership (government, mission, private, industry) and facility class (hospital, health centre, clinic, other). The ninth and last facility, a tuberculosis clinic, was selected by opportunistic sampling.

Interviews were conducted in a private space in health facilities during working hours. The study and interview dates were advertised beforehand. Study participants had to be full-time or at least part-time employees at a healthcare facility and over 18 years of age. Participants were approached using convenience sampling of those staff present at the facility during the time of the interviews. Care was taken to avoid coercion, handpicking or selection of favoured staff as respondents by facility directors.

In total, a sample of 34 nurses, technicians and other healthcare providers participated in the study, shown in Table 1. Only one physician participated in the study, however, and this physician did not complete the interview and withdrew consent.

**Table 1:** Respondents by cadre*

Health worker type	Frequency	%	% in Swaziland workforce**
Nurse	12	35.3	32%

Orderly	6	17.6	13%

Health support staff (driver, cleaner, receptionist, intern)	5	15%	33%

Laboratory technician	3	8.8	2%

Radiology technician	2	5.9	1%

Pharmacist	2	5.9	1%

Environmental health officer	1	2.9	3%

Health administrator	1	2.9	7%

Rehabilitation technician	1	2.9	< 1%

Social worker	1	2.9	No data

*Total*	*34*	*100*	

*59% female; average age 43 years; 53% health clinics, 32% hospital, 15% health centres; 38% governmental, 29% mission, 18% non-governmental organization, 15% private; 41% employed less than five years and 6% over 20 years

** Source: African Health Workforce Observatory (2009) Human Resources for Health Country Profile Swaziland. World Health Organization, Geneva

### Data analysis

Interviews were transcribed in English by an independent translator. Initial coding, analysis and reporting were conducted by a research assistant and one of the principal investigators using qualitative data-analysis software, with final review by the two principal investigators. Content analysis was used to arrange the data into major themes and subthemes. Preliminary results were shared with partners in-country during a two-day results validation workshop in Swaziland in 2008. During this period, feedback was obtained from partners and used to adjust, refine and finalize the results.

### Ethical considerations

To assure confidentiality and anonymity of information, complete anonymity of respondents was maintained in data sheets or transcripts, HIV status information was requested in general terms only (not specific to the respondents), and interviews were conducted in private. Informed consent was obtained by signature and permission to tape oral interviews. The survey protocol was approved by the Swaziland Research Ethics Committee in the MOHSW.

## Results

### HIV/AIDS in the work environment

Table 2 shows general responses from selected survey variables concerning HIV/AIDS in the work environment and treatment and prevention services (next section) by sex.

**Table 2:** General results relevant survey variables

Survey variable	% women	% men	% total
*HIV/AIDS in the work environment*			

States that HIV affected a health worker who they knew personally	85	57	74

Have seen colleagues miss work due to HIV	60	43	53

Have seen colleagues miss work due to sick family member	60	36	50

Thinks healthcare workers are open about their status	25	7	18

As a healthcare worker, likes to know their HIV/AIDS status	95	93	94

Finds that there is a need for special services for health workers	85	93	88

*Prevention and treatment services*			

Received training on HIV/AIDS prevention and control	60	43	53

Received training in post-exposure prophylaxis (PEP)	70	57	63

Always practices universal precautions at workplace when handling patients	70	64	68

Finds it "very" to "somewhat easy" to access and obtain PEP	75	79	77

Finds that other health workers might encounter more problems in accessing PEP than themselves	30	36	32

As can be seen, HIV was experienced as a significant work environment problem, particularly as observed by women health workers. About three-quarters of the respondents reported having personally known a colleague affected by HIV/AIDS and about half of the respondents noted that they had seen colleagues missing work due to a personal or family member's HIV infection. Health workers further estimated the number of days a month that colleagues missed coming to work as a result of personal or a family member's HIV infection in the past month to be, on average, 10 days (weighted average of midpoints of the categories: 1-6 days, n = 9; 7-13 days, n = 9; 14-20 days, n = 5; 21-30, n = 2). In interview narratives, respondents observed poor performance and absenteeism (getting sick, being unable to work in a normal way, and taking on light duties and special assignments) among fellow health workers. Another common observation was increased stress and financial concerns as a result of having a family member ill from HIV.

### Prevention and treatment services

Table 2 also shows that the perceived general availability of HIV prevention and treatment services did not appear to be a major barrier to accessing treatment for both sexes. Most of the respondents indicated practicing (or wanting to practice) universal precautions and infection prevention methods. Basic supplies were found to be mostly available and used properly for this purpose, with the exception of government-supported hospitals. About one-third of the sample said that sometimes, materials were lacking, with the exception of latex gloves, mostly because materials were not the right size or were out of stock. A lack of proper waste disposal was observed (incinerators are less available in health clinics).

**Figure 1:**
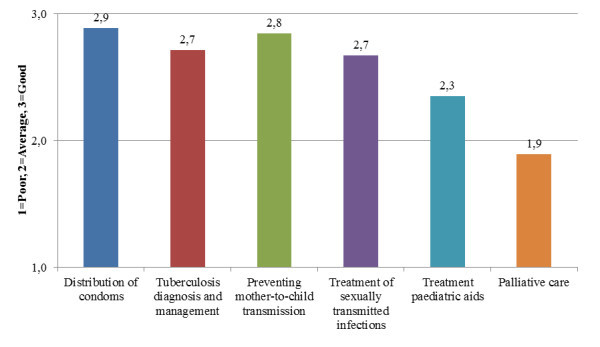
**Availability of prevention and treatment services**. 1 = poor, 2 = average, 3 = good.

Further, health workers judged the availability of services as good for most of the following categories: voluntary counselling and testing, ART, condom provision, tuberculosis diagnosis and treatment, prevention of mother to child transmission services, sexually transmitted infection treatment, and paediatric ART (see Figure 1).

Perceived service availability did not seem to differ by gender, but positive availability estimates did increase by age and duration of employment. Peer support groups and home-based care services were reported to be available to about half of the respondents, although respondents at the same site gave conflicting responses to whether these services were provided. Further, health workers seemed generally unsure about what community-based services were available.

**Figure 2:**
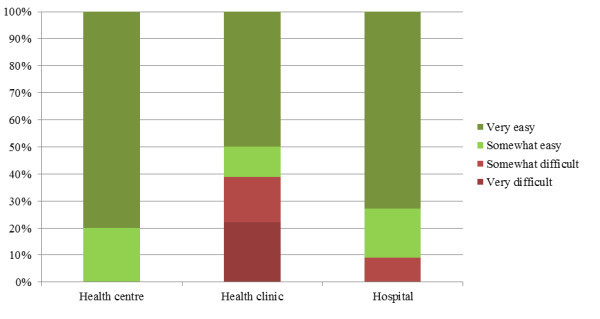
**Access to PEP by type of facility**.

**Figure 3:**
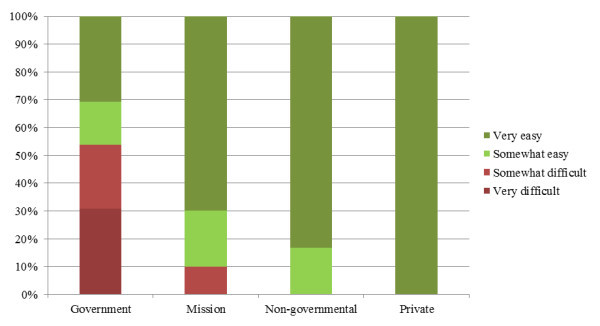
**Access to PEP by facility ownership**.

One out of four respondents mentioned that access to PEP was somewhat to very difficult. Reasons given included ignorance, limited availability at night and on weekends, expense of purchasing antiretroviral drugs, lack of accreditation, and insufficient providers and unavailability of ARVs. Access appeared most difficult in health clinics (see Figure 2) and government facilities (see Figure 3). Nurses and nurse assistants expressed more difficulty obtaining access than technicians, pharmacists and support staff. No differences were found regarding gender.

While we did not ask directly whether participants had been tested or were HIV positive, with the exception of two respondents, all interviewed health workers mentioned wanting to know their own HIV status (Table 2). When asked about the major Swaziland stand-alone health worker clinic, the Wellness Center [22], 85% of the respondents mentioned having heard of the centre, yet only 40% - mostly respondents younger than 40 working at health centres run by non-governmental organizations - mentioned having visited it "for minor issues".

### Self-stigmatization and the HIV/AIDS status boundary

Most respondents agreed that "health workers" were generally *not *open about their status: this observation was shared by 75% of the female and 93% of the male health workers. Moreover, many cited examples of colleagues denying their HIV infection. While official sanction or loss of employment was not given as a reason not to be open about HIV status, our respondents cited as major barriers fear of negative judgements and labels of being promiscuous or diseased being given to them by other health workers, as well as by the general public. The generally dismissive culture of censorship within the health profession was mentioned as contributing to health workers' "self-stigmatization". As a 30-year-old male nurse explained:

Most of the health workers are still not free about HIV issues. You can get that from their comments about HIV. So it makes the others scared to share their ideas or feelings about HIV. They comment negatively about HIV. It is like health workers are giving themselves self-stigmatization on HIV, so that is why it is difficult.

Self-stigmatization was exacerbated as health workers strongly felt a sense of failure or professional embarrassment for contracting an infection that they felt they should have had the knowledge to avoid. According to a 34-year-old female nurse:

I think they feel like the general public will be surprised to know that I am HIV positive. And I have all the information. I have accessibility to everything - condoms, drugs. If they know that I am HIV positive, they will be like, "wow. How can you tell us to do this and you are not doing it?"

Health workers indicated that they perceived that they were at *less *risk and therefore less likely to take steps to protect themselves from sexual HIV acquisition or find opportunities to test themselves and seek needed care. They indicated that they felt that messages about HIV were for "the general public" and that efforts to reduce stigma were not targeted at health workers. A 34-year-old female nurse noted about this situation:

The general public has a decreased impact of stigma, and health workers have a high impact of stigma because probably there was no one who was saying to them, "this is yours." It was always for "them"; HIV is not for "us". We are learning, we are reading books, we are getting informed. Not for us; it is for our patients. That made them ignore themselves, that they need to take HIV as part of them.

Reasons given by health workers for undergoing HIV/AIDS testing did not explicitly include concern with their *own *status, but instead focused on the needs of the patients and general public, and the health worker as a role model. Respondents mentioned that being tested was a chance to better understand the testing process that their patients undergo, improve their skills in recognizing patients' experiences, serve as role models, and protect patients from acquiring HIV infection from the healthcare provider.

The notion that HIV/AIDS is "not for health workers" appeared to reinforce the fear that one's positive status is wrongly attributed by others to personal risk behaviour as opposed to professional exposure. As a 23-year-old male nursing student noted:

Maybe my problem will be that if I found that I am positive after doing that test I will be afraid, because they won't think that it was due to this professional injury that I have just sustained, maybe it's my long-time behaviour, and so that can be a barrier problem to me to access that.

### The need for privacy and confidentiality

Responses from one of the vignette scenarios, shown in Box 1, emphasize self-stigmatization while pointing out another major issue creating barriers to care: mistrust in true privacy and confidentiality of test results. This vignette focused on what health workers would imagine to be the most likely behaviour of others based on their own experience.

Imagine a female nurse comes to a colleague and friend and reveals

that she has recently discovered that she is HIV positive. She says

she does not wish to register at the public ART clinic as she knows

*the providers there*.

### Vignette #1

Respondents were asked what they would advise the HIV-infected nurse to do, and a follow-up question asked what they thought she would actually do. The majority of respondents advised the hypothetical nurse to go to another facility or the Swaziland Wellness Center to avoid encountering known patients or colleagues and feel more comfortable as a result. This emphasizes the importance of privacy and confidentiality as a most significant factor in the general advice given. As one 34-year-old female nurse explained, this issue is particularly relevant to the densely connected social network of Swaziland health workers:

We are a small country. We have been to only two nursing school[s] so it is quite impossible not to know anyone in a health-providing facility. There is no way you cannot find someone you know in any facility, not unless you just take a tour in all the facilities and that might be very expensive.

It was mentioned often that there would be very few places where she would not know some of the health workers. This was echoed by many respondents who advised her to stay at her local clinic, as there was really no place she could go for complete anonymity.

Despite this, a third of all respondents thought that the HIV-infected nurse in the scenario would eventually access care. Some said that she would be forced to address the issue when her illness became more symptomatic. Many thought that her own education about the benefits of care and comfort with HIV would help her. Others thought that she would derive benefit from receiving comfort and sympathy from colleagues. However, the majority of the respondents said that it would be unlikely that the health worker in Vignette 1 would seek care openly, regardless of whether they had advised her to do this or not. According to respondents, providers at the clinic would talk about the nurse to others if she came to visit for testing or care. Moreover, most respondents indicated that the nurse would assume that the providers would do so *even *if in reality the provider actually did protect her privacy, a norm of expectations commonly shared among health workers. In other words, imagined lack of confidentiality appeared to be a driving force for not seeking needed care.

Many respondents thought that it would be unlikely for the nurse to access care due to her own fears. One respondent also mentioned that professional embarrassment related to her status might be used against her by the clinic providers when professional conflicts arose. Revealing status to supervisors or colleagues with official roles of providing HIV care was not a first choice by many. If care had to be sought, respondents said, an exclusive room or special staff and procedures clearly ensuring privacy and confidentiality were needed, yet were often lacking. In many cases, however, what the nurse would actually do was thought to be influenced by her own attitudes and the attitudes of the providers she encountered, particularly related to stigma.

## Discussion

The finding that HIV had affected work performance is not surprising and has been described by others [12]. Respondents, however, gave novel insight into the HIV-related stigma they suffer. A very strong theme was the unique sense of professional embarrassment that health workers feel about contracting HIV, combined with perceived (or imagined) lack of confidentiality, leading to a strong form of self-stigmatization. Health workers appear to be policing the boundary between their own professional identity as HIV-free social models and the identity of their patients and the general public, who are seen as vulnerable to the impacts of the disease.

This observed situation conforms to a process referred to as "othering" in the social sciences. Otherness refers to the tendency to perceive another group or person as different and not the same as "me" or "we". Otherness is typically defined as a socio-cultural process by which a dominant group defines and reinforces its power by labelling those who do not fit the model as "other" [23]. The characteristics of that dominant group are often taken for granted, unexamined, and used as the norm that provides the standard for judging the other. Health workers defined themselves as having a secure, positive identity of being "HIV/AIDS free" in opposition to the general public which, stigmatized as "the other", is expected to be vulnerable to HIV/AIDS.

From this perspective, the health worker's fear of being classified as one of "the others" is arguably related to a perceived and professionally imposed sense of morality in which there is no place for an HIV-positive status. Members of the health worker profession are expected to be HIV/AIDS free, and this boundary is explicitly patrolled by health workers themselves. Embodied rituals of the profession, such as prevention behaviours, help reinforce this immediate boundary between the health worker population and the "others", from both internal and external perspectives.

While othering typically leaves the "other" vulnerable, in this case, it is the group patrolling its own morality that might be on the losing end because resulting gaps in health worker care remain invisible and unaddressed. The reïfication of the health worker as free of HIV is a denial of a history in which the vulnerability of the health worker to the epidemic is significant [24]. As general HIV prevention messages leave out this health worker vulnerability, the boundary-making process is affirmed from the outside, as well.

The suggestion of a self-stigmatizing health workforce suggests that overcoming the fear surrounding HIV may be even greater in health workers than other groups. It should be noted that while we did not directly ask, no respondent gave examples of actual negative consequences that HIV-infected health workers had faced as a result of being HIV positive. This may be because openly HIV-positive health workers are extremely rare. We did not address how this self-stigmatization correlated with stigmatizing behaviour *toward *HIV patients, which could also be an effect.

In this context, it is not surprising to find a call for improved privacy and confidentiality among health workers when seeking care. This concern seems also sensible in a regional community like Swaziland where most health workers (especially nurses) know one another. When a worker did reveal an HIV-positive status, this was typically done based on a trusted rather than professional (care-related) relationship. Given this, if anonymity cannot be achieved for HIV-positive health workers, access could be improved by allowing health workers to choose, from a list of designated providers, the person they feel most comfortable having as a provider. Furthermore, special services made available should be of high quality, free of charge, easily accessible by public transport, with expanded night and weekend hours of operation, staffed by professionals, with a non-bureaucratic system of identification that is not discriminatory, and not solely focusing on HIV to avoid discriminatory concerns.

The study has several limitations. Opportunistic sampling using a novel measurement tool might have biased results towards those with strong views. Further, staff members who were absent may have different views than those who were present at work as absenteeism may be related to HIV status, HIV in their family, or other factors. Generalizability of the findings is therefore limited. Vignettes were chosen over asking respondents directly about their own HIV status, which may have limited accuracy, despite improvements in accuracy and wording after field testing.

Further, despite intensive training, data collectors were nurses and not professional researchers, which may have also impacted on results. This impact could be both negative (such as biased responses due to interviewer technique or the seniority of one of the nurse interviewers) and positive (such as greater understanding, comfort and openness than if interviewers had been outside researchers) [25]. While respondents were diverse, the lack of participation of physicians (only one presented to be interviewed and then withdrew consent) may partly be the result of including nurses in the research team, although physicians comprise only 1% of the Swazi health workforce. The theme of a perceived need for privacy in this study might have been exacerbated by Swaziland's small size.

## Conclusions

Results illustrate the profound impact that HIV has on the health workforce in Swaziland. Respondents emphasized the importance of private and confidential treatment and offered previously undocumented insights into the impacts of professional "othering" and self-stigmatization that surrounds HIV-infected health workers. Prevention messages about HIV should address these fears specifically, i.e., that acknowledging that HIV can happen to anyone regardless of their training and may also happen to health workers. This humanization of health workers is urgent. Importantly, this information comes directly from health workers themselves, describing their own beliefs and opinions and gathered through a study designed with their input.

## Competing interests

The authors declare that they have no competing interests.

## Authors' contributions

DHdV carried out the research and survey design, led the data analysis and drafted the manuscript. SG conceived the study, participated in its design and coordination and helped draft the manuscript. MM, BC and TD read and approved the final manuscript. MM, BC and TD participated in research and survey design, conducted the data collection, provided input in data analysis and reviewed the manuscript. All authors have read and approved the final version of this manuscript.
